# Through the Cracks: Diagnostic Dilemma of Cystic Hypersecretory Hyperplasia in a Limited Breast Sample

**DOI:** 10.7759/cureus.93132

**Published:** 2025-09-24

**Authors:** Hijab Shah, Zouheir Maarouf, Lekha Potti, Ioannis Michalakis, Fergus Young

**Affiliations:** 1 Histopathology, North Cumbria Integrated Care (NCIC) NHS Foundation Trust, Carlisle, GBR; 2 Breast Radiology, North Cumbria Integrated Care (NCIC) NHS Foundation Trust, Carlisle, GBR; 3 Breast Oncoplastic Surgery, North Cumbria Integrated Care (NCIC) NHS Foundation Trust, Carlisle, GBR

**Keywords:** breast pathology, breast screening, management, needle core biopsy, pathology

## Abstract

We describe a diagnostically challenging case of cystic hypersecretory hyperplasia (CHH), an exceptionally rare benign breast lesion that is often difficult to distinguish from more aggressive pathologies. The patient, an asymptomatic woman in her sixth decade, was identified during routine breast screening. Imaging evaluation revealed conflicting findings: mammography demonstrated features that raised suspicion for malignancy, whereas ultrasound appearances were more compatible with a benign process. Clinical assessment offered no additional clarity, underscoring the complexity of the diagnostic workup. Histological confirmation proved equally challenging. The initial needle core biopsy specimen provided insufficient material for a definitive diagnosis, necessitating a second biopsy. Only after this additional sampling was the lesion accurately classified as CHH. This case illustrates the diagnostic limitations of small-volume tissue sampling, particularly in rare entities with subtle histological features.

Although CHH typically lacks cytological atypia, its recognized association with atypical and malignant variants-including cystic hypersecretory carcinoma-supports its categorization as a lesion of uncertain malignant potential. Consequently, surgical excision remains the preferred management approach, even in the absence of overt atypia, to exclude coexistent or progressive disease.

This report emphasizes the critical role of multimodal imaging, repeated histopathological evaluation when necessary, and a cautious surgical strategy to ensure accurate diagnosis and safe patient management in rare breast lesions with ambiguous presentations.

## Introduction

Cystic hypersecretory hyperplasia (CHH) represents an exceptionally rare and poorly understood breast lesion, characterized by its uncertain etiology and incompletely elucidated pathogenesis. From a prognostic standpoint, this entity is generally considered benign when identified in isolation. However, its clinical significance lies in the documented association with both cystic hypersecretory hyperplasia with atypia and cystic hypersecretory carcinoma (CHC), entities that are believed to exist along a pathological continuum of progressive morphological alterations [1]. Recognition of this continuum is crucial, as it underscores the potential for lesions initially classified as benign to coexist with, or evolve into, atypical or frankly malignant counterparts.

Although the UK National Health Service Breast Screening (NHS BS) multidisciplinary working group has developed robust guidelines that outline diagnostic categorization and management strategies for a wide spectrum of breast lesions-including those classified as lesions of uncertain malignant potential (B3)-these recommendations remain vague when applied to exceedingly rare entities such as CHH, when encountered in core needle biopsy specimens [2]. The absence of explicit guidance introduces a significant degree of uncertainty into clinical decision-making, thereby posing a challenge for both pathologists and multidisciplinary teams responsible for patient management.

The diagnostic process is further complicated by the intrinsic limitations of core needle biopsy sampling, which, by nature, yields a restricted volume of tissue. In some instances, the diagnostic material retrieved may be scant, leading to an incomplete representation of the lesion’s architecture. This is particularly problematic when evaluating rare entities such as CHH, which display histopathological features that can mimic those of more common benign conditions, most notably fibrocystic change. Fibrocystic change is a frequent, entirely benign breast lesion that typically warrants a B2 classification and requires no further clinical intervention. However, failure to recognize the subtle but diagnostically critical histological distinctions between CHH and fibrocystic change on limited core biopsy material may result in a missed diagnosis. Such an oversight carries clinical implications, given the well-established association of CHH with a spectrum of more sinister lesions, including borderline and malignant forms.

For these reasons, meticulous histopathological assessment is imperative when evaluating core needle biopsies that demonstrate features suggestive of cystic and secretory alterations. Pathologists must remain alert to the subtle morphological clues that differentiate CHH from its benign mimics to ensure accurate classification and appropriate clinical management.

## Case presentation

A woman in her early 60s, a lifelong non-smoker, presented for routine screening as part of the United Kingdom National Health Service Breast Screening Programme (NHS BSP). Her past medical history was significant for biopsy-proven lung adenocarcinoma diagnosed two years earlier, which had been appropriately managed. She was postmenopausal, had no history of hormone replacement therapy (HRT), and reported no family history of breast or other carcinomas.

During screening mammography, an irregular, well-defined mass measuring approximately 30 mm was identified in the upper outer quadrant of the left breast. The imaging features were highly suspicious for malignancy and were therefore categorized as M5 according to Breast Imaging Reporting and Data System (BI-RADS) equivalent used in the NHS BSP. Subsequent ultrasound examination revealed a hypoechoic mass measuring 24 mm in maximum dimension, which was interpreted as probably benign (U3), creating a radiological discordance. Imaging of the axilla was unremarkable, with no evidence of lymphadenopathy. A marker clip was deployed, and a core needle biopsy of the lesion was performed. On clinical examination, the breast findings were indeterminate and categorized as P3.

**Figure 1:**
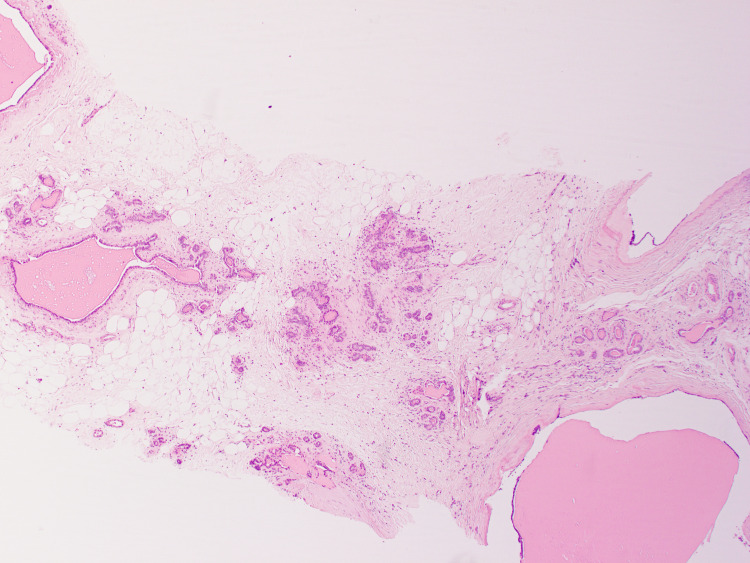
Benign breast tissue was seen with dilated ducts containing intraluminal eosinophilic secretions with no significant fibrosis or inflammation in the background stroma.

**Figure 2:**
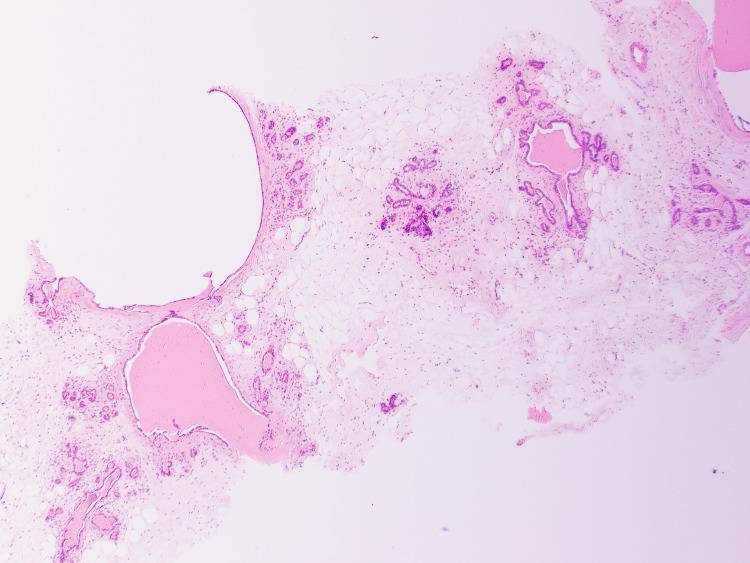
Dilated breast ducts with eosinophilic secretions.

Histopathological assessment of the initial core biopsy demonstrated benign breast tissue with dilated ducts containing intraluminal eosinophilic secretions. The background stroma exhibited no significant fibrosis or inflammatory infiltrate. Importantly, there was no evidence of epithelial atypia, hyperplasia, or malignancy. Based on these findings, the lesion was diagnosed as fibrocystic change (B2 - benign) (Figures 1, 2).

However, given the significant radiology-pathology discordance between a mammographic impression of malignancy (M5) and a benign histological diagnosis (B2), the case was escalated for discussion at the multidisciplinary screening breast meeting (MDT). The consensus was to perform a repeat core biopsy for further evaluation.

**Figure 3:**
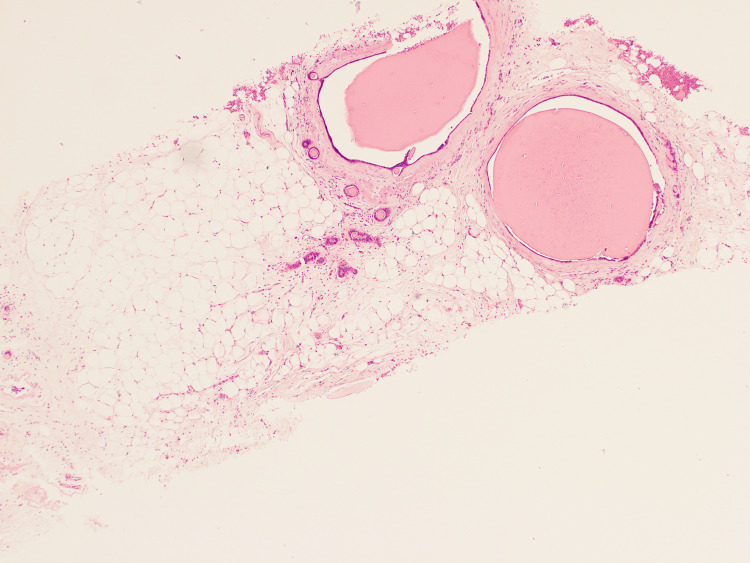
Dilated cystic ducts with intraluminal homogenous thyroid-like colloidal material and artefactual shrinking and retraction of the cyst contents.

The second biopsy revealed multiple dilated cystic spaces containing intraluminal homogeneous, eosinophilic, thyroid-like colloid material, along with artefactual retraction and shrinkage of cyst contents. The cysts were lined by a simple, flattened epithelium, and the surrounding stroma showed minimal fibrosis without significant inflammatory changes (Figure 3). No epithelial atypia or malignancy was identified.

The case was reviewed in detail by local histopathologists with a special interest in breast pathology. Although some features overlapped with those of fibrocystic change, the overall morphology raised concern for CHH, a rare entity associated with a spectrum of lesions ranging from CHH with atypia to CHC.

Given the diagnostic uncertainty and potential clinical implications, the case was re-discussed at the breast screening MDT, where the pathologist highlighted the absence of clear management protocols for CHH within the current NHS BSP guidelines. In view of the lesion’s classification as B3 (lesion of uncertain malignant potential) without atypia, an excision biopsy was recommended to ensure complete evaluation.

**Figure 4:**
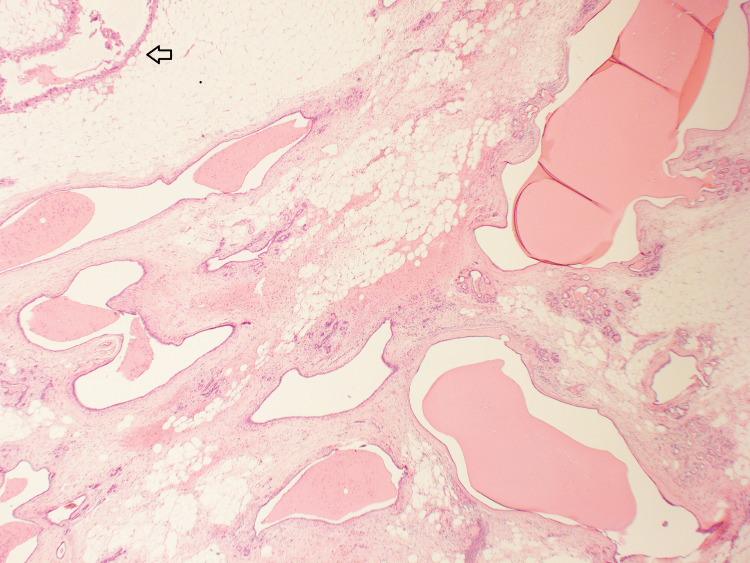
Cystic hypersecretory hyperplasia (previous biopsy site is also seen, marked with arrow).

A diagnostic surgical excision of the lesion was subsequently performed. Histological examination confirmed the previous core biopsy findings of cystic hypersecretory hyperplasia, with characteristic cystic spaces containing thick, eosinophilic secretions and lined by attenuated epithelium (Figure 4). There was no evidence of atypia, hyperplasia, or invasive malignancy.

## Discussion

CHH is an uncommon and under-recognized benign breast lesion, most frequently reported in women between the ages of 38 and 62 years [1]. Although it is generally regarded as a benign entity, its rarity and potential association with atypical and malignant counterparts underscore the importance of accurate diagnosis and appropriate management. CHH may present as a solitary lesion or, less commonly, as part of a multifocal process involving different regions of the breast. The exact pathogenesis remains incompletely understood, primarily due to the limited number of reported cases, though some authors suggest that CHH may represent a hyperplastic response associated with hormonal influence or aberrant secretory activity. Interestingly, CHH is often observed in a background of lactational-type changes, even in non-pregnant, non-lactating women, suggesting an underlying hormonal component in its development [3].

Clinical presentation is variable. While some patients present with a palpable breast mass or nipple discharge, a significant proportion remain asymptomatic and are detected incidentally during routine screening [4]. This was the scenario in our case, where the lesion was identified through the NHS Breast Screening Programme in an otherwise asymptomatic patient.

Radiological findings are nonspecific and can mimic both benign and malignant processes. Mammography may demonstrate asymmetric densities, circumscribed or partially circumscribed masses, or occasionally suspicious calcifications. On ultrasound, CHH often appears as a cystic or mixed echogenic lesion, while MRI may show mass or non-mass enhancement patterns [5]. This variability contributes to the frequent radiology-pathology discordance observed in such cases, further complicating clinical decision-making.

From a histopathological standpoint, CHH is characterized by multiple dilated ducts and cysts containing dense, eosinophilic, colloid-like secretions, which may exhibit artefactual cracking and shrinkage. The cysts are typically lined by bland cuboidal or low-columnar epithelium without evidence of cytological or architectural atypia, distinguishing CHH from its more sinister counterparts [6]. Occasional reactive inflammation may be observed, usually secondary to cyst rupture. The surrounding breast tissue often demonstrates lactational changes, such as dilated terminal duct lobular units lined by hobnail cells with cytoplasmic vacuolization, enlarged nuclei, and prominent nucleoli [7].

Although CHH itself is benign, its clinical significance lies in its well-documented association with cystic hypersecretory hyperplasia with atypia and CHC. The latter is an extremely rare variant of ductal carcinoma, with approximately 20 cases reported in the literature, almost all of which have arisen in a background of CHH or its atypical form [6]. This observation strongly suggests that CHH may act as a precursor lesion within a morphological continuum progressing from benign to malignant disease, though the precise biological mechanism remains unclear.

Management of CHH identified on core needle biopsy poses a significant challenge. Core biopsies, by their nature, provide limited tissue, which may not adequately represent the entire lesion. Given the potential coexistence of CHH with atypical or malignant components, a diagnosis of CHH on core biopsy is typically categorized as B3 (lesion of uncertain malignant potential). In accordance with best practice, diagnostic surgical excision is strongly recommended to exclude the presence of more aggressive pathology [7]. Excision biopsy is generally considered curative when no atypia or malignancy is present, but failure to excise the lesion could risk missing an associated CHC, which has significant prognostic implications [4].

## Conclusions

This case reinforces several important points including the necessity for a high index of suspicion in cases with imaging-pathology discordance and the pivotal role of multidisciplinary team (MDT) discussion in guiding management decisions for rare lesions without standardized protocols. It also highlights the importance of surgical excision for definitive diagnosis and to rule out associated atypical or malignant changes in biopsy proven cases of cystic hypersecretory hyperplasia. Further research and case studies are also needed to clarify the natural history of CHH.
